# Comparison of 6 Mortality Risk Scores for Prediction of 1-Year Mortality Risk in Older Adults With Multimorbidity

**DOI:** 10.1001/jamanetworkopen.2022.23911

**Published:** 2022-07-27

**Authors:** Claudio Schneider, Carole E. Aubert, Cinzia Del Giovane, Jacques D. Donzé, Viktoria Gastens, Douglas C. Bauer, Manuel R. Blum, Olivia Dalleur, Séverine Henrard, Wilma Knol, Denis O’Mahony, Denis Curtin, Sei J. Lee, Drahomir Aujesky, Nicolas Rodondi, Martin Feller

**Affiliations:** 1 Department of General Internal Medicine, Inselspital, Bern University Hospital, University of Bern, Bern, Switzerland; 2 Institute of Primary Health Care, University of Bern, Bern, Switzerland; 3 Department of Medicine, Neuchâtel Hospital Network, Neuchâtel, Switzerland; 4 Division of Internal Medicine, Lausanne University Hospital, Lausanne, Switzerland; 5 Department of General Internal Medicine, Brigham and Women’s Hospital, Harvard Medical School, Boston, Massachusetts; 6 Graduate School for Health Sciences, University of Bern, Bern, Switzerland; 7 Departments of Medicine and Epidemiology and Biostatistics, University of California, San Francisco, San Francisco; 8 Cliniques Universitaires Saint-Luc, Université Catholique de Louvain, Louvain, Belgium; 9 Louvain Drug Research Institute–Clinical Pharmacy Research Group, Université Catholique de Louvain, Louvain, Belgium; 10 Institute of Health and Society, Université Catholique de Louvain, Louvain, Belgium; 11 Department of Geriatric Medicine and Expertise Centre Pharmacotherapy in Old Persons, University Medical Center Utrecht, Utrecht University, Utrecht, the Netherlands; 12 Department of Medicine (Geriatrics) University College Cork and Cork University Hospital, Cork, Republic of Ireland; 13 Division of Geriatrics, University of California, San Francisco, San Francisco

## Abstract

**Importance:**

The most appropriate therapy for older adults with multimorbidity may depend on life expectancy (ie, mortality risk), and several scores have been developed to predict 1-year mortality risk. However, often, these mortality risk scores have not been externally validated in large sample sizes, and a head-to-head comparison in a prospective contemporary cohort is lacking.

**Objective:**

To prospectively compare the performance of 6 scores in predicting the 1-year mortality risk in hospitalized older adults with multimorbidity.

**Design, Setting, and Participants:**

This prognostic study analyzed data of participants in the OPERAM (Optimising Therapy to Prevent Avoidable Hospital Admissions in Multimorbid Older People) trial, which was conducted between December 1, 2016, and October 31, 2018, in surgical and nonsurgical departments of 4 university-based hospitals in Louvain, Belgium; Utrecht, the Netherlands; Cork, Republic of Ireland; and Bern, Switzerland. Eligible participants in the OPERAM trial had multimorbidity (≥3 coexisting chronic diseases), were aged 70 years or older, had polypharmacy (≥5 long-term medications), and were admitted to a participating ward. Data were analyzed from April 1 to September 30, 2020.

**Main Outcomes and Measures:**

The outcome of interest was any-cause death occurring in the first year of inclusion in the OPERAM trial. Overall performance, discrimination, and calibration of the following 6 scores were assessed: Burden of Illness Score for Elderly Persons, CARING (Cancer, Admissions ≥2, Residence in a nursing home, Intensive care unit admit with multiorgan failure, ≥2 Noncancer hospice guidelines) Criteria, Charlson Comorbidity Index, Gagné Index, Levine Index, and Walter Index. These scores were assessed using the following measures: Brier score (0 indicates perfect overall performance and 0.25 indicates a noninformative model); C-statistic and 95% CI; Hosmer-Lemeshow goodness-of-fit test and calibration plots; and sensitivity, specificity, and positive and negative predictive values.

**Results:**

The 1879 patients in the study had a median (IQR) age of 79 (74-84) years and 835 were women (44.4%). The median (IQR) number of chronic diseases was 11 (8-16). Within 1 year, 375 participants (20.0%) died. Brier scores ranged from 0.16 (Gagné Index) to 0.24 (Burden of Illness Score for Elderly Persons). C-statistic values ranged from 0.62 (95% CI, 0.59-0.65) for Charlson Comorbidity Index to 0.69 (95% CI, 0.66-0.72) for the Walter Index. Calibration was good for the Gagné Index and moderate for other mortality risk scores.

**Conclusions and Relevance:**

Results of this prognostic study suggest that all 6 of the 1-year mortality risk scores examined had moderate prognostic performance, discriminatory power, and calibration in a large cohort of hospitalized older adults with multimorbidity. Overall, none of these mortality risk scores outperformed the others, and thus none could be recommended for use in daily clinical practice.

## Key Points

**Question:**

What is the validity of 6 widely used scores for predicting the 1-year mortality risk in older adults with multimorbidity?

**Findings:**

In this prognostic study of 1879 older adults with multimorbidity, all 6 of the 1-year mortality risk scores examined showed moderate prognostic performance, discriminatory power, and calibration.

**Meaning:**

Findings of this study suggest that none of the examined mortality risk scores outperformed the others, and thus they could not be recommended for use in daily clinical practice.

## Teaser

This prognostic study evaluates the overall performance, discriminatory power, and calibration of the Burden of Illness Score for Elderly Persons, CARING Criteria, Charlson Comorbidity Index, Gagné Index, Levine Index, and Walter Index.

## Introduction

In Europe, more than 60% of adults aged 65 years or older have multimorbidity.^1^ Patients with multimorbidity are often treated for each disease separately, applying single disease–focused guidelines, without accounting for other comorbidities. Therefore, patients with multiple chronic diseases are often prescribed multiple drugs for each disease with little regard to potentially cumulative, harmful consequences of the multiple medications. Most evidence on risk reduction with medical treatment is extrapolated from randomized clinical trials involving younger populations and selected groups of relatively healthy older individuals.^2^ A meta-analysis of studies of the treatment of hypertension in adults aged 80 years or older suggested that the benefit (36% decrease in relative stroke risk) might be offset by adverse effects given that the overall risk of death increased by 14% under antihypertensive treatment.^3^ Patients who use 5 or more drugs (polypharmacy) have a higher risk for adverse drug events, drug-drug interactions, lower quality of life, and fatal outcomes.^4,5^ There is little evidence that multimorbidity in older adults should be treated in the same way as multimorbidity in younger and healthier individuals.^2^

Many patients with multimorbidity and a high mortality risk are exposed to the potential harms of preventive medications that provide little chance of benefit.^6^ Discontinuing potentially inappropriate medications (ie, deprescribing) may be beneficial in these cases.^7^ However, decisions to deprescribe are often challenging because of the difficulty of making an accurate prognosis. Accurate mortality risk prediction could inform clinical decision-making and enable physicians to align treatments to the condition, preferences, and prognosis of their patients.

Many mortality risk scores have been developed for use in older adults in different settings.^8^ However, these mortality risk scores have not been externally validated in large sample sizes, and a head-to-head comparison in a contemporary prospective cohort is lacking.^8^ Thus, there is currently no consensus on which mortality risk score performs best in older adults with multimorbidity. In this prognostic study, we aimed to prospectively compare the performance of 6 scores in predicting the 1-year mortality risk in hospitalized older adults with multimorbidity.

## Methods

### Study Setting and Participants

This prognostic study analyzed data from participants in the OPERAM (Optimising Therapy to Prevent Avoidable Hospital Admissions in Multimorbid Older People) trial.^9,10^ The OPERAM trial was approved by the local ethics committees at each site. Participants gave informed consent to participate in the OPERAM trial and its substudies. Approval for the present substudy was waived by the Ethics Commission of the Canton of Bern because participants had already agreed to the use of their data in the OPERAM trial substudies. We followed the Transparent Reporting of a Multivariable Prediction Model for Individual Prognosis or Diagnosis (TRIPOD) reporting guideline.^11^

The OPERAM trial was a multicenter, cluster randomized clinical trial that examined the effect of a structured medication review intervention (compared with standard care) on drug-related hospital admissions. The trial recruited participants and was conducted between December 1, 2016, and October 31, 2018, in surgical and nonsurgical departments of 4 university-based hospitals in Bern, Switzerland; Cork, Republic of Ireland; Louvain, Belgium; and Utrecht, the Netherlands. Eligible individuals were those with multimorbidity (≥3 coexisting chronic diseases lasting at least 6 months), aged 70 years or older, with polypharmacy (≥5 long-term medications), and admitted to a participating ward in these 4 hospitals. All OPERAM trial participants (the validation cohort) were followed up for 12 months (with the last follow-up completed in October 2019). Further details on and results of this trial design have been published.^9,10^

### Mortality Risk Scores 

After a review of existing mortality risk scores, we selected the scores on the basis of 2 criteria: (1) the score had to predict 1-year mortality risk and (2) the information or variables required to calculate the score had to be available in the OPERAM trial.^10^ We identified 6 mortality risk scores that met these 2 criteria: Burden of Illness Score for Elderly Persons (BISEP; score range: 0-7, with the highest score indicating 74% mortality risk),^12^ CARING (Cancer, Admissions ≥2, Residence in a nursing home, Intensive care unit admit with multiorgan failure, ≥2 Noncancer hospice guidelines) Criteria (score range: 0-44, with the highest score indicating 49% mortality risk),^13^ Charlson Comorbidity Index (CCI; score range: 0-37, with the highest score indicating 85% mortality risk),^14^ Gagné Index (score range: –2 to 26 with the highest score indicating 46.8% mortality risk),^15^ Levine Index (score range: 0-11, with the highest score indicating 46% mortality risk),^16^ and Walter Index (score range: 0-20, with the highest score indicating 68% mortality risk).^17^ The Gagné Index predicts mortality risk for community-dwelling patients^15^; all other scores predict mortality risk for hospitalized patients.^12,13,14,16,17^

Trained research nurses used standardized forms to collect patient baseline information on hospital admission. Trained physicians transferred diagnoses into *International Statistical Classification of Diseases and Related Health Problems, Tenth Revision* codes. The characteristics of participants in the original studies^12,13,14,15,16,17^ (the development cohort) that evaluated the six 1-year mortality risk scores are described in eTable 1 in the Supplement.

### Outcome Measure

The outcome of interest was death from any cause occurring within the first year of inclusion in the OPERAM trial. Death during hospitalization was captured from the medical records, and death after discharge was assessed by trained research nurses through telephone follow-up with general practitioners. The last available medical documentation (eg, discharge letter, general practitioner record) was collected.^9^

### Statistical Analysis

Each mortality risk score was calculated using computer-based algorithms in Stata, version 16.0 (StataCorp LLC). If diagnoses were necessary to calculate a mortality risk score, we used the widely used coding algorithm for comorbidities developed by Quan et al.^18^ For each variable, we reported the number (proportion) of participants with missing data. In accordance with the clinical application of prognostic models,^19^ we assumed for the primary analysis that missing values were normal (eg, “not living in nursing home” when this information was missing for some participants). In a sensitivity analysis, we calculated the mortality risk scores after multiple imputation for the missing albumin level and nursing home data.

We assessed the performance of the 6 mortality risk scores using the following measures: (1) Brier score, a measure of overall performance; (2) C-statistic with 95% CI, a measure of discrimination; (3) Hosmer-Lemeshow goodness-of-fit-test and calibration plots, a measure of calibration; and (4) sensitivity, specificity, and positive and negative predictive values, measures of 1-year mortality risk of 20% or greater. In addition, we restricted the sensitivity analysis to participants who did not die during the index hospitalization.

The Brier score ranges from 0 (indicating perfect overall performance) to 0.25 (indicating noninformative model) and simultaneously addresses discrimination (the concentration of the predictive distribution) and calibration (the statistical consistency between the predicted probability and the observations).^20^ The C-statistic indicates how well a score discerns the risk of death (ie, participants with a higher mortality risk according to the score are more likely to die during follow-up than participants with a lower mortality risk according to the score); however, it does not indicate how accurate the absolute mortality risk is. The C-statistic value ranges from 0.5 (indicating no discriminatory power) to 1.0 (indicating perfect discriminatory power). Specifically, C-statistic values of 0.9 or higher indicate excellent; 0.8 to 0.89, very good; 0.7 to 0.79, good; 0.6 to 0.69, moderate; and 0.5 to 0.59, poor discriminatory power.^21^ Furthermore, we performed a pairwise comparison of the receiver operating characteristic curves of each mortality risk score using the DeLong method.^22^

Ideally, observed deaths are perfectly predicted by the mortality risk score. The null hypothesis of the Hosmer-Lemeshow goodness-of-fit test is that the assessed mortality risk score predicts death correctly. Because the power of the Hosmer-Lemeshow goodness-of-fit tests increases with the sample size, small discrepancies between a predicted and observed death are likely to lead to the rejection of the null hypothesis with large sample sizes, even if such discrepancies are irrelevant for the scope of the mortality risk score.^23^ Therefore, we reran the Hosmer-Lemeshow goodness-of-fit test in a sensitivity analyses using a random subsample of 100 participants to check which of the mortality risk scores would perform best and become insignificant. Furthermore, we used the method proposed by Yourman and colleagues^8^ for visual judgment of the calibration plots. Yourman and colleagues^8^ considered 10% or greater point difference between predicted and observed mortality to be poor calibration and less than 10% point difference to be good calibration. We defined persons with 20% or greater predicted 1-year mortality risk as having high risk of mortality, which corresponded to the mean 1-year mortality risk among the study population,^10^ in the absence of a widely accepted cutoff in the literature.^24^

All statistical tests were 2-sided, and *P* < .05 was considered statistically significant. We used Stata, version 16.0 for all analyses (StataCorp LLC). Data were analyzed from April 1 to September 30, 2020.

## Results

### Study Sample

Of the 2008 participants in the OPERAM trial, 119 withdrew from the trial before or after the final assessment and 10 were lost to follow-up. The final study sample comprised 1879 participants, with a median (IQR) age of 79.3 (74.4-84.4) years and 835 women (44.4%) and 1044 men (55.6%). The median (IQR) number of diagnoses was 11.0 (8.0-16.0), and the median (IQR) number of medications was 11.0 (8.0-14.0). More than one-fourth of participants (n = 520 [27.7%]) had a current cancer diagnosis or experienced cancer. Within 1 year, 375 participants (20.0%) died. Additional baseline characteristics are shown in Table 1. For comparison with the participants in the OPERAM trial, we provided the baseline characteristics of the participants in the development cohorts of the 6 mortality risk scores in eTable 2 in the Supplement. Except for the Gagné Index, the observed mortality risk per risk stratum of each mortality risk score was generally lower in the validation cohort (OPERAM trial participants) compared with the development cohorts (Table 2).

**Table 1.** Baseline Characteristics of Participants in the OPERAM Trial^a^

Characteristic	Participants, No. (%)
No. of participants	1879
Sex	
Female	835 (44.4)
Male	1044 (55.6)
Age, median (IQR), y	79.3 (74.4-84.4)
Length of hospital stay, median (IQR), d	8.5 (6.0-14.0)
No. of diagnoses, median (IQR)	11.0 (8.0-16.0)
No. of drugs, median (IQR)	11.0 (8.0-14.0)
Current or experienced cancer diagnosis	520 (27.7)
Living in nursing home	96 (5.9)
Discharge to nursing home	155 (8.4)
≥2 Admissions in past year	445 (23.8)
Laboratory values, median (IQR)	
Albumin, g/dL	3.3 (2.8-3.7)
Creatinine, mg/dL	1.1 (0.8-1.5)
Activity of daily living, dependent	
Bathing	596 (32.0)
Feeding	150 (8.0)
Dressing	524 (28.0)
Toileting and hygiene	338 (18.1)
Transferring	329 (17.6)
Mobility	405 (21.7)
Study site	
Switzerland	805 (42.8)
Belgium	338 (18.0)
The Netherlands	406 (21.6)
Republic of Ireland	330 (17.6)

Abbreviation: OPERAM, Optimising Therapy to Prevent Avoidable Hospital Admissions in Multimorbid Older People.

SI conversion factor: To convert albumin levels to grams per liter, multiply by 10; creatinine levels to micromoles per liter, multiply by 76.25.

^a^ Information about missing data is provided in eTable 7 in the Supplement.

**Table 2.** Mortality Risk in the Validation Cohort vs Development Cohorts

Score and score points	Validation (OPERAM) cohort		Development cohorts	
	No. (%)	Mortality risk (95% CI), %	No. (%)	Mortality risk (95% CI), %
BISEP, No.^a^	1879		525	
0-1	496 (26.4)	10.1 (7.6-13.1)	249 (47.4)	8.4 (6.3-9.7)
2	368 (19.6)	14.1 (10.7-18.1)	103 (19.6)	24.3 (19.8-28.2)
3	413 (22.0)	22.8 (18.8-27.1)	86 (16.4)	51.2 (45.6-56.4)
≥4	602 (32.0)	29.7 (26.1-33.6)	87 (16.6)	73.6 (69.3-78.7)
CARING Criteria, No.^b^^,^^c^	1879		873	
≤4	957 (50.9)	14.0 (11.9-16.4)	NA	<18
5-12	392 (20.9)	20.7 (16.8-25.0)	NA	18.0-48.9
≥13	530 (28.2)	30.2 (26.3-34.3)	NA	≥49
CCI, No.^d^	1879		459	
0	243 (12.9)	10.3 (6.8-14.8)	181 (39.4)	12 (9.6-14.4)
1-2	714 (38.0)	15.1 (12.6-18.0)	125 (27.2)	26 (22.1-29.9)
3-4	613 (32.6)	23.7 (20.3-27.2)	71 (15.5)	52 (46.1-57.9)
≥5	309 (16.4)	31.4 (26.3-36.9)	82 (17.9)	85 (81.1-88.9)
Gagné Index, No.^b^^,^^e^	1879		12 0679	
<0	142 (7.6)	7.9 (4.0-13.6)	NA	2.4 (2.2-2.6)
0	304 (16.2)	11.2 (7.9-15.3)	NA	3.6 (3.4-3.8)
1	332 (17.7)	15.1 (11.4-19.4)	NA	5.1 (4.9-5.4)
2	279 (14.8)	19.5 (14.9-24.7)	NA	7.8 (7.4-8.3)
3	237 (12.6)	19.1 (14.3-24.8)	NA	11.3 (10.7-12.0)
4	216 (11.5)	20.8 (15.6-26.9)	NA	14.6 (13.8-15.5)
5	159 (8.5)	33.1 (25.8-41.1)	NA	20.1 (18.9-21.4)
6	81 (4.3)	33.7 (24.4-43.9)	NA	24.9 (23.3-26.5)
7	56 (3.0)	29.4 (17.5-43.8)	NA	29.5 (24.4-31.6)
8-9	49 (2.6)	46.3 (32.6-60.4)	NA	36.5 (34.4-38.7)
>9	24 (1.3)	48.3 (29.4-67.5)	NA	46.8 (43.4-50.1)
Levine Index, No.^f^	1879		2739	
0-1	36 (1.9)	2.8 (0.1-14.5)	799 (29.2)	13.8 (11.6-16.4)
2	216 (11.5)	5.6 (2.9-9.5)	719 (26.3)	18.1 (15.5-21.1)
3	561 (29.9)	13.7 (11.0-16.9)	563 (20.6)	32.0 (28.3-36.1)
≥4	1066 (56.7)	26.7 (24.1-29.5)	647 (23.6)	46.2 (42.5-50.2)
Walter Index, No.^g^	1879		1494	
0-1	349 (18.6)	6.3 (4.0-9.4)	356 (23.8)	12.9 (9.9-16.9)
2-3	522 (27.8)	14.9 (12.0-18.3)	382 (25.5)	20.2 (16.5-24.6)
4-6	701 (37.3)	22.1 (19.1-25.4)	475 (31.8)	37.0 (33.0-41.7)
≥7	307 (16.3)	39.1 (33.6-44.8)	282 (18.9)	68.4 (63.2-74.1)

Abbreviations: BISEP, Burden of Illness Score for Elderly Persons; CARING, Cancer, Admissions ≥2, Residence in a nursing home, Intensive care unit admit with multiorgan failure, ≥2 Noncancer hospice guidelines; CCI, Charlson Comorbidity Index; NA, not applicable; OPERAM, Optimising Therapy to Prevent Avoidable Hospital Admissions in Multimorbid Older People.

^a^ BISEP score range: 0-7, with the highest score indicating 74% mortality risk.

^b^ Number of participants per risk stratum was not reported in the original publication.

^c^ CARING Criteria score range: 0-44, with the highest score indicating 49% mortality risk.

^d^ CCI score range: 0-37, with the highest score indicating 85% mortality risk.

^e^ Gagné Index score range: –2 to 26 with the highest score indicating 46.8% mortality risk.

^f^ Levine Index score range: 0-11, with the highest score indicating 46% mortality risk.

^g^ Walter Index score range: 0-20, with the highest score indicating 68% mortality risk.

### Performance of the Mortality Risk Scores

Overall, the Gagné Index (Brier score, 0.16), the CARING Criteria (Brier score: 0.17), and the Walter Index (Brier score, 0.17) performed best followed by the Levine Index (Brier score, 0.19) (Table 3). The CCI (Brier score, 0.23) and BISEP (Brier score, 0.24) were close to noninformative in predicting 1-year mortality risk in this population. All 6 mortality risk scores had moderate discriminatory power with a C-statistic value ranging from a CCI of 0.62 (95% CI, 0.59-0.65) to a Walter Index of 0.69 (95% CI, 0.66-0.72) (Table 3); receiver operating characteristic curves are shown in eFigure 1 in the Supplement). In the pairwise comparison of the receiver operating characteristic curves, Walter Index (C-statistic value, 0.69; 95% CI, 0.66-0.72) outperformed the other mortality risk scores (all *P* < .05). Furthermore, the BISEP (C-statistic value, 0.65; 95% CI, 0.62-0.68), Gagné Index (C-statistic value, 0.65; 95% CI, 0.62-0.68), and Levine Index (C-statistic value, 0.66; 95% CI, 0.63-0.69) performed significantly better than the CCI (C-statistic value, 0.62; 95% CI, 0.59-0.65; *P* < .05) (eTable 3 in the Supplement).

**Table 3.** Overall Performance, Discriminatory Ability, and Calibration of 6 Mortality Risk Scores

Score	Overall performance using Brier score^**b**^	Discriminatory ability		Calibration using Hosmer-Lemeshow goodness-of-fit test^**a**^	
		C-statistic	95% CI	χ^2^	*P* value
BISEP	0.24	0.65	(0.62-0.68)	767.5	<.01
CARING Criteria	0.17	0.64	(0.61-0.67)	104.7	<.01
CCI	0.23	0.62	(0.59-0.65)	938.4	<.01
Gagné Index	0.16	0.65	(0.62-0.68)	89.8	<.01
Levine Index	0.19	0.66	(0.63-0.69)	271.7	<.01
Walter Index	0.17	0.69	(0.66-0.72)	206.8	<.01

Abbreviations: BISEP, Burden of Illness Score for Elderly Persons; CARING, Cancer, Admissions ≥2, Residence in a nursing home, Intensive care unit admit with multiorgan failure, ≥2 Noncancer hospice guidelines; CCI, Charlson Comorbidity Index.

^a^ The null hypothesis of the Hosmer-Lemeshow goodness-of-fit test is that the assessed score predicts death correctly. Thus, a significant *P* value indicates poor calibration.

^b^ Brier score ranged from 0 (perfect overall performance) to 0.25 (noninformative model).

The visual analysis revealed that the Gagné Index had good calibration over the full score. The BISEP, CARING Criteria, CCI, and Walter Index showed poor calibration at extremes when mortality risk was greater than 20% (Figure). The Hosmer-Lemeshow goodness-of-fit test was significant for every mortality risk score, with all *P* < .01 for χ^2^ ranging from 89.8 to 767.5, formally indicating poor calibration (Table 3). The calibration plot is shown in eFigure 2 in the Supplement. In a sensitivity analysis, we reran the Hosmer-Lemeshow goodness-of-fit test for each mortality risk score on a random subsample of 100 participants. The Gagné Index had a *P* > .99 for χ^2^ of 1.2, indicating good calibration. For the other mortality risk scores, the *P* value remained significant or, in the case of the CARING Criteria, close to significant with a *P* = .06 for χ^2^ of 7.5, indicating poor overall calibration (eTable 4 in the Supplement).

**Figure.**
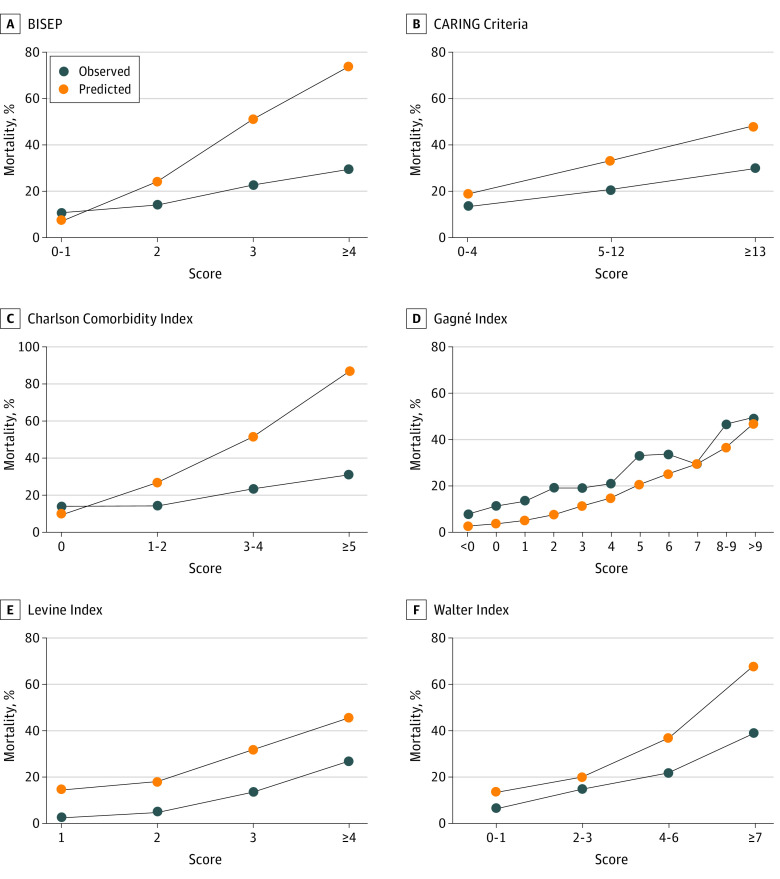
Calibration Curves for 1-Year Mortality Each predicted or observed mortality (y-axis) was predicted for 1 risk category group of participants. Risk categories were created by grouping a certain amount of achieved scores (x-axis). The closer the observed or predicted curves, the better the calibration. BISEP indicates Burden of Illness Score for Elderly Persons; CARING, Cancer, Admissions ≥2, Residence in a nursing home, Intensive care unit admit with multiorgan failure, ≥2 Noncancer hospice guidelines.

We calculated sensitivity, specificity, and positive and negative predictive values of each mortality risk score to predict a 20% or greater 1-year mortality risk. The Levine Index had the highest sensitivity with 97% (95% CI, 94%-98%), at a cost of a low specificity of 16% (95% CI, 14%-18%). On the other hand, the Gagné Index had a low sensitivity of 48% (95% CI, 43%-53%) and an acceptable specificity of 73% (95% CI, 71%-75%). Positive predictive values ranged from 21% (95% CI, 19%-24%) for CCI to 31% (95% CI, 27%-35%) for the Gagné Index. Negative predictive values ranged from 85% (95% CI, 83%-87%) for the Gagné Index to 95% (95% CI, 91%-97%) for the Levine Index. The results are summarized in Table 4.

**Table 4.** Measure of Performance to Predict 1-Year Mortality Risk of 20% or Greater

	% (95% CI)			
	Sensitivity	Specificity	PPV	NPV
BISEP	87 (83-90)	30 (27-32)	24 (21-26)	90 (87-92)
CARING Criteria	64 (59-69)	55 (52-57)	26 (23-29)	86 (84-88)
CCI	93 (90-96)	15 (13-16)	21 (19-24)	90 (85-93)
Gagné Index	48 (43-53)	73 (71-75)	31 (27-35)	85 (83-87)
Levine Index	97 (94-98)	16 (14-18)	22 (20-24)	95 (91-97)
Walter Index	94 (91-96)	22 (20-24)	23 (21-25)	94 (91-96)

Abbreviations: BISEP, Burden of Illness Score for Elderly Persons; CARING, Cancer, Admissions ≥2, Residence in a nursing home, Intensive care unit admit with multiorgan failure, ≥2 Noncancer hospice guidelines; CCI, Charlson Comorbidity Index; NPV, negative predictive value; PPV, positive predictive value.

The results remained similar in the sensitivity analysis when we calculated the mortality risk scores after multiple imputation for the missing albumin level and nursing home data (eTable 5 in the Supplement). When we restricted the analysis to participants who survived the index hospitalization (during which 71 participants died), the results also remained robust (eTable 6 in the Supplement).

## Discussion

This external validation of six 1-year mortality risk scores in a large cohort of hospitalized older patients with multimorbidity found that all 6 scores had a moderate prognostic performance, discriminatory power, and calibration, with none of the scores outperforming the others. The CCI, as the most used mortality risk score, had a rather low performance compared with the other scores.^25^ These results suggest that it remains unclear whether 1-year mortality risk in older adults could be accurately predicted with a yet-to-be-developed score or whether such a score could, at best, be only 1 piece of a more comprehensive estimation of life expectancy.

To our knowledge, the only similar study that evaluated prognostic indices in hospitalized older patients tested 5 mortality risk scores (Walter Index, Levine Index, CARING Criteria, Silver Code, BISEP) in a small (N = 100) population of older adults (mean age, 86 years) in 2012 in Italy.^26^ The authors reported similar C-statistic values, ranging from 0.51 (Silver Code) to 0.72 (BISEP), but found better calibration of the BISEP and the Walter Index than the other mortality risk scores. A possible explanation for why all of the mortality risk scores performed rather unsatisfyingly may be the distinction between multimorbidity and comorbidity.^27,28^ Most etiological research thus far has focused on a given chronic condition (eg, chronic kidney disease) and has assessed comorbidities to describe the burden of illness in the population under study. In such a hierarchical context with a common index disease and associated comorbidities, it is conceivable that mortality risk scores might perform better. However, in older adults with multimorbidity (such as the present cohort) who do not share a common disease along with comorbidities and for whom information about the severity of each disease is lacking, the quest for a simple and accurate 1-year mortality risk score may remain futile.

An important, yet unresolved question concerns the cutoff to define a high 1-year mortality risk. In the present study, we defined this cutoff as 20% or greater mortality risk because it was the mean 1-year mortality risk in the population. The Gagné Index (as the best performing mortality risk score in this analysis) had a positive predictive value of 31% and a negative predictive value of 85%, illustrating that the examined mortality risk scores might be useful in identifying patients for whom longer-term preventive measures may likely be beneficial. Although every mortality risk score is currently only 1 piece of an assessment, which is complemented by the practical knowledge of the experienced physician, it seems that none of the six mortality risk scores we examined performs well enough to be adopted in daily clinical practice to guide therapeutic decisions.

Except for the Gagné Index, all of the other mortality risk scores predicted a substantially higher mortality risk than was actually observed in this older population with multimorbidity. This observation was even more pronounced with increasing mortality risk (Figure). Examining the baseline characteristics of the participants in the original studies (eTable 2 in the Supplement), we did not find apparent differences between the validation cohort (participants in the OPERAM trial) and the development cohorts that could explain the higher mortality risk in the original studies. Of note, much information about the health status of the participants in the development cohorts was not provided, hindering a more detailed comparison. A potential explanation for the discrepancy in the lower-than-predicted mortality is that all of the original studies were conducted in the US. In contrast, the validation cohort originated from 4 Western European countries, whose health care systems with mandatory health insurance may be a factor in the lower mortality risk in older adults with multimorbidity.^29,30,31^ Another possible explanation is that the OPERAM trial was conducted at least 15 years after the original studies were performed. Medical care for older adults with multimorbidity may have improved in the past 15 years. This hypothesis is supported by the Gagné Index, the only mortality risk score that did not overestimate mortality risk in the validation cohort, being the only score that was developed in the 21st century.

### Limitations

This study has several limitations. First, albumin levels that would be required to calculate the BISEP and Walter Index were not assessed systematically in all participants. Therefore, 717 participants (38.2%) had missing data for albumin levels . Furthermore, we had no information for 226 participants (12.0%) on whether they were living in a nursing home. We retained all participants with missing information on these variables in the main analysis, assuming normal values for albumin level and that participants did not live in a nursing home.^19^ After multiple imputations for missing albumin level and nursing home data, we found that the results were similar. Second, the calibration using the Hosmer-Lemeshow goodness-of-fit test was significant for all mortality risk scores, formally indicating bad calibration and contrasting the visual judgment of good calibration for Gagné Index and all other indices when mortality risk was low. Yet, the Hosmer-Lemeshow goodness-of-fit test was originally developed through simulation studies of hypothetical samples of 200 observations,^32^ whereas the sample size of the present study was 1879. The null hypothesis assumes perfect fit, an assumption that becomes more and more problematic with increasing sample size.^23,33,34^ There were attempts to set rules to optimize statistical power and to obtain meaningful results with the test by performing an adequate selection of the sample size.^35^ Thus, in the sensitivity analysis on a random subsample of 100 participants, the Gagné Index was the only mortality risk score that was no longer significant. Assuming that the Hosmer-Lemeshow goodness-of-fit test is overpowered in the large sample size, this finding with an insignificant Gagné Index in a smaller sample size suggests that the index has good calibration and is in line with the visual judgment.

Third, although *International Classification of Diseases* diagnosis codes were available for every participant, we did not know when someone had a primary diagnosis of cancer, which was needed to calculate the CARING Criteria. To calculate the CARING Criteria, we assumed a primary diagnosis of cancer whenever a participant had a known diagnosis of cancer. We cannot exclude the possibility that this inaccuracy was associated with the performance of the CARING Criteria in this study. Fourth, none of the 6 mortality risk scores captured disability and frailty. As the prognostic implications of these 2 concepts become increasingly important, we cannot overlook that the integration of these 2 concepts would improve the 1-year mortality risk prediction in older adults with multimorbidity. Fifth, we could not deny that the participants who could provide consent were likely at a lower mortality risk than those who could not. This observation may be a potential bias and explain why the mortality risk scores predicted a higher mortality risk.

## Conclusions

This comparison of 6 mortality risk scores found that all scores had moderate overall prognostic performance, discriminatory power, and calibration. Overall, not one of these scores outperformed the others, and thus not one could be recommended for use in daily clinical practice.

## Notes

Supplement. **eFigure 1.** Area Under the Receiver Operating Characteristic Curves for 1-Year Mortality **eFigure 2.** Score Calibration Plot for 1-Year Mortality **eTable 1.** Characteristics of the Six Evaluated 1-Year Mortality Scores **eTable 2.** Participant Characteristics **eTable 3.** Pairwise Comparison of the ROC Curves **eTable 4.** Calibration of the Six Scores Analyzed With the Brier Score and the Hosmer-Lemeshow Chi Square Test With the Hosmer-Lemeshow Chi Square Test Run on a Subsample **eTable 5.** Sensitivity Analysis. Overall Performance, Discriminatory Ability and Calibration of the Six Scores Analyzed With the Brier Score, C-Statistics and Hosmer-Lemeshow Goodness of Fit Test Using Multiple Imputation for Missing Data in Variables for Albumin Values and Nursing Home Residence **eTable 6.** Sensitivity Analysis. Overall Performance, Discriminatory Ability and Calibration of the Six Scores Analyzed With the Brier Score, C-Statistics and Hosmer-Lemeshow Goodness of Fit Test After Excluding All 71 Death During Hospitalization **eTable 7.** OPERAM Patient Characteristics at Baseline With Information About Missing Data
eReferences
