# Supplementary material for: Comparison of 6 Mortality Risk Scores for Prediction of 1-Year Mortality Risk in Older Adults With Multimorbidity
Source: JAMA Netw Open. 2022 Jul 27;5(7):e2223911. doi: 10.1001/jamanetworkopen.2022.23911 (PMC9331084; doi:10.1001/jamanetworkopen.2022.23911)
Supplement: Supplement. — eFigure 1. Area Under the Receiver Operating Characteristic Curves for 1-Year Mortality eFigure 2. Score Calibration Plot for 1-Year Mortality eTable 1. Characteristics of the Six Evaluated 1-Year Mortality Scores eTable 2. Participant Characteristics eTable 3. Pairwise Comparison of the ROC Curves eTable 4. Calibration of the Six Scores Analyzed With the Brier Score and the Hosmer-Lemeshow Chi Square Test With the Hosmer-Lemeshow Chi Square Test Run on a Subsample eTable 5. Sensitivity Analysis. Overall Performance, Discriminatory Ability and Calibration of the Six Scores Analyzed With the Brier Score, C-Statistics and Hosmer-Lemeshow Goodness of Fit Test Using Multiple Imputation for Missing Data in Variables for Albumin Values and Nursing Home Residence eTable 6. Sensitivity Analysis. Overall Performance, Discriminatory Ability and Calibration of the Six Scores Analyzed With the Brier Score, C-Statistics and Hosmer-Lemeshow Goodness of Fit Test After Excluding All 71 Death During Hospitalization eTable 7. OPERAM Patient Characteristics at Baseline With Information About Missing Data eReferences [file jamanetwopen-e2223911-s001.pdf]

Supplemental Online Content

Schneider C, Aubert CE, Del Giovane C, et al. Comparison of 6 mortality risk scores for prediction of 1-year mortality risk in older adults with multimorbidity. *JAMA Netw Open*. 2022;5(7):e2223911. doi:10.1001/jamanetworkopen.2022.23911

eFigure 1. Area Under the Receiver Operating Characteristic Curves for 1-Year Mortality

eFigure 2. Score Calibration Plot for 1-Year Mortality

eTable 1. Characteristics of the Six Evaluated 1-Year Mortality Scores

eTable 2. Participant Characteristics

eTable 3. Pairwise Comparison of the ROC Curves

eTable 4. Calibration of the Six Scores Analyzed With the Brier Score and the Hosmer-Lemeshow Chi Square Test With the Hosmer-Lemeshow Chi Square Test Run on a Subsample

eTable 5. Sensitivity Analysis. Overall Performance, Discriminatory Ability and Calibration of the Six Scores Analyzed With the Brier Score, C-Statistics and Hosmer-Lemeshow Goodness of Fit Test Using Multiple Imputation for Missing Data in Variables for Albumin Values and Nursing Home Residence

eTable 6. Sensitivity Analysis. Overall Performance, Discriminatory Ability and Calibration of the Six Scores Analyzed With the Brier Score, C-Statistics and Hosmer-Lemeshow Goodness of Fit Test After Excluding All 71 Death During Hospitalization

eTable 7. OPERAM Patient Characteristics at Baseline With Information About Missing Data

eReferences

This supplemental material has been provided by the authors to give readers additional information about their work.

© 2022 Schneider C et al. *JAMA Network Open.*

eFigure 1. Area Under the Receiver Operating Characteristic Curves for 1-Year Mortality.

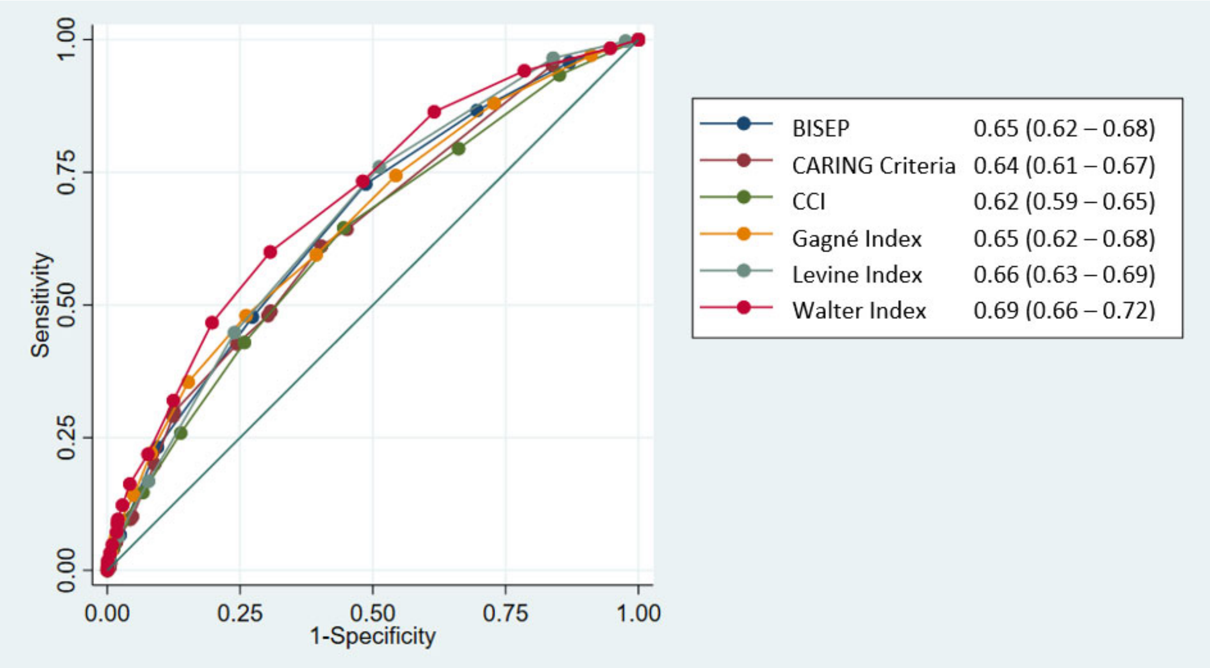

C-Statistic for each 1-year mortality score calculated by score points, with 95% confidence in parenthesis. Values ≥ 0.9 indicate excellent, 0.8 – 0.89 very good, 0.7 – 0.79 good, 0.6 – 0.69 moderate and 0.5 – 0.59 poor discriminatory power.[21] The C-statistic significantly (p-value < 0.01) differ with the CCI performing worst and the Walter Index performing best.

Abbreviations: AUC, area under the receiver operating characteristic curve; BISEP, Burden of illness Score; CCI, Charlson Comorbidity Index.

© 2022 Schneider C et al. *JAMA Network Open.*

eFigure 2. Score Calibration Plot for 1-Year Mortality

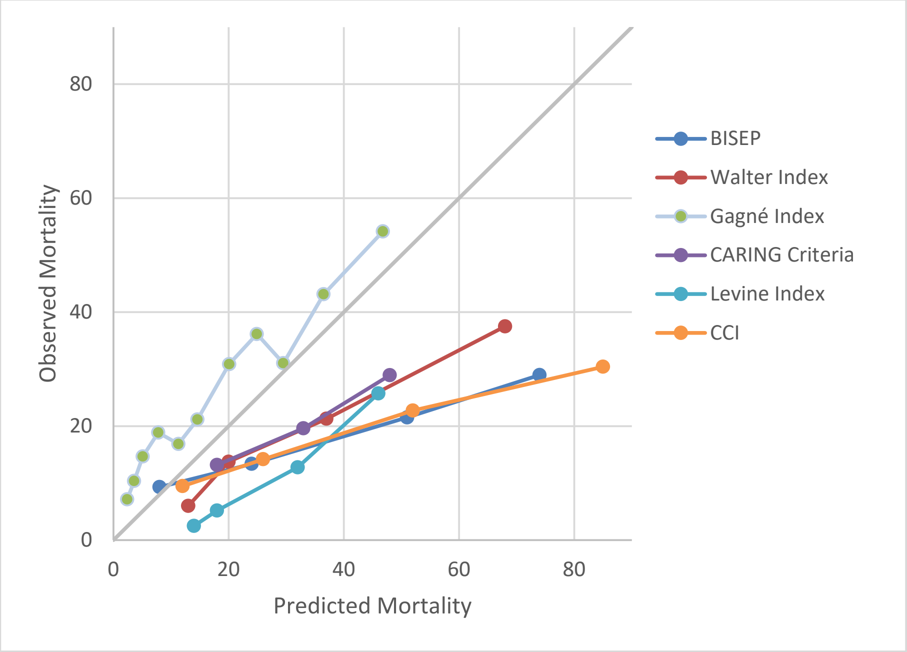

Calibration plot with predicted mortality on the x-axis and the observed mortality on the y-axis. If a model calibrates well, it is close to the 45° line of perfect fit.
Abbreviations: BISEP, Burden of illness Score for Elderly Persons; CCI, Charlson Comorbidity Index.

© 2022 Schneider C et al. *JAMA Network Open.*

eTable 1. Characteristics of the Six Evaluated 1-Year Mortality Scores

Index	Cohort	Items	Points	Predicted mortality [%] (total points)
**Burden of**	Development:	Critical diagnosis		5
**Illness Score**	n = 525	(maximal 3 points in this section		(0-1 point)
**for Elderly**	Age ≥ 70 y	cumulative)		
**Persons**	Mean age 79 y	- Lymphoma or leukemia	3	16
**(BISEP)**	Hospitalized patients	- Acute renal failure	2	(2 points)
		- Metastatic cancer	2	
Inouye et al.	External validation:	- Localized cancer	2	32
2003[12]	n = 1246	- Stroke	1	(3 points)
	Age ≥ 65 y	- Congestive heart failure	1	
	Mean age 81 y	- Chronic lung disease	1	61
	Nursing home patients	- Chronic renal failure	1	(> 3 points)
		- Diabetes mellitus	1	
		(with end-organ damage)		
		- Pneumonia	1	
		Albumin ≤ 35 g/l	1	
		Creatinine > 115 umol/l	1	
		Dementia	1	
		Need help / is unable to walk	1	
**CARING**	Development:	Age 55 - 65	1	<18
**Criteria**	n = 435	66 - 75	2	(< 5 points)
	Hospitalized patients	≥ 75	3	
Fischer et al.				18-48
2006[13]	Internal Validation:	Cancer as primary diagnosis	10	(5-12 points)
	n = 438	Admissions ≥ 2	3	
	Hospitalized patients	Residence in a nursing home	3	>48
		Intensive care unit with	10	(>12 points)
	Overall:	multiorgan failure		
	Mean age 63 y	Meet non-cancer hospice guidelines (≥2)	12	
**Charlson**	Development:	Metastatic solid tumor	6	12
**Comorbidity**	n = 559	HIV/AIDS	6	(0 points)
**Index**	Hospitalized patients	Moderate to severe liver disease	3	
		Lymphoma	2	26
Charlson et al.		Leukaemia	2	(1-2 point(s))
1987[14]	External validation:	Any tumor	2	
	n = 685	Diabetes with end organ damage	2	52
	Hospitalized patients	Moderate to severe renal disease	2	(3-4 points)
	with proven primary	Hemiplegia	2	
	carcinoma of the	Myocardial infarct	1	85
	breast	Congestive heart failure	1	(> 4 points)
		Peripheral vascular disease	1	
		Chronic pulmonary disease	1	
		Cerebrovascular disease	1	
		Dementia	1	
		Connective tissue disease	1	
		Ulcer disease	1	
		Mild liver disease	1	
**Gagne Index**	Development:	Metastatic cancer	5	2.4
	n = 120679	Congestive heart failure	2	(<0 points)
Gagne et al.	Age > 65 y	Dementia	2	3.6
2011[15]	Mean age 80 y	Renal failure	2	(0 points)
	Community dwelling	Weight loss	2	5.1
		Hemiplegia	1	(1 point)
	External validation:	Alcohol abuse	1	7.8
	n = 123855	Any tumour	1	(2 points)
	Mean age 79 y	Cardiac arrhythmias	1	11.3
	Community dwelling	Chronic pulmonary disease	1	(3 points)
		Coagulopathy	1	14.6
		Complicated diabetes	1	(4 points)
		Deficiency anaemias	1	20.1
		Fluid and electrolyte disorders	1	(5 points)
		Liver disease	1	24.9
		Peripheral vascular disorder	1	(6 points)
		Psychosis	1	29.5
		Pulmonary circulation disorders	1	(7 points)
		HIV/AIDS	1	36.5
		Hypertension	1	(8-9 points)
				46.8
				(>9 points)

© 2022 Schneider C et al. *JAMA Network Open.*

**Levine Index**	Development:	Age	70-74 y	1	14
	n = 2739		≥ 75 y	2	(0-1 point)
Levine et al.	Mean age 78 y				
2007[16]	Hospitalized	Discharge to nursing home		1	24
	patients	Length of stay	≥ 5 days	1	(2 points)
		Heart failure		1	
	Internal validation:	Peripheral Vascular Disease		1	30
	n = 3543	Dementia		1	(3 points)
	Mean age 78 y	Renal disease		1	
	Hospitalized	Hematologic and solid malignancy		1	42
	Patients	Metastatic cancer		2	(>3 points)
**Walter Index**	Development:	Gender	Male	1	13
	n = 1495		Female	2	(0-1 point)
Walter et al.	Age > 70 y				
2001[17]	Mean age 81 y	ADLs	Bath		20
	Hospitalized patients	on discharge	Dressing		(2-3 points)
			Transfer (if 1-4)	4	
	External validation:		Toilet (if 5)	5	37
	n = 1427		Eating		(4-6 points)
	Mean age 79 y				
	Hospitalized patients	Congestive heart failure		2	68
					(>6 points)
		Cancer	No metastasis	3	
			With metastasis	6	
		Creatinine on admission (> 265umol/l)		2	
		Albumin on admission	30-34 g/l	1	
			< 30 g/l	2	

Abbreviations: BISEP, Burden of illness Score for Elderly Persons; CCI, Charlson Comorbidity Index; n, Number of participants; y, years.

© 2022 Schneider C et al. *JAMA Network Open.*

eTable 2. Participant Characteristics

N<br>Characteristics, mean (range, % or SD)	OPERAM<br>1879	BISEP[12]<br>525	CARING[13]<br>873	CCI*[14]<br>559	Gagné[15]<br>120,679	Levine[16]<br>2739	Walter[17]<br>1495
Age[y]	80 (70-100)	79 (70-98)	63 (13)		80 (7.3)	78 (8.3)	81 (7)
Female sex, [n] (%)	835 (44.4%)	294 (56.0%)	(2%)		(83.4%)	1733 (63%)	1004 (67%)
Length of stay [days]	11.8 (1-155)						
Charlson >= 2, [n] (%)		372 (71.0%)					
Number of diagnoses	13.0 (1-58)				18.0 (IQR 11-27)		
Number of medications	11.4 (1-36)				8.0 (IQR 5-12)		
Living in nursing home	96 (5.8%)	37 (7.1%)	26 (3%)		9.1%		
Discharge to nursing home	155 (8.4%)					415 (15%)	452 (30%)
**Death, n (%)**							
Death within one year	375 (20.0%)	154 (29.3%)	229 (26.2%)		10,769 (8.9%)	415 (15.2%)	492 (32.9%)

Abbreviations: BISEP, Burden of illness Score for Elderly Persons; CCI Charlson Comorbidity Index, IQR, interquartile range.

* None of the information could be found in the published literature.

eTable 3. Pairwise Comparison of the ROC Curves

	**BISEP**	**CARING Criteria**	**Charlson Comorbidity Index**	**Gagné Index**	**Levine Index**	**Walter Index**
**BISEP**	x	0.4395	**0.0178**	0.9472	0.6290	**0.0015**
**CARING Criteria**	0.4395	x	0.3267	0.5101	0.1932	**0.0003**
**Charlson Comorbidity Index**	**0.0178**	0.3267	x	**0.0076**	**0.0030**	**0.0000**
**Gagné Index**	0.9472	0.5101	**0.0076**	x	0.5042	**0.0039**
**Levine Index**	0.6290	0.1932	0.0030	0.5042	x	**0.0130**
**Walter Index**	**0.0015**	**0.0003**	**0.0000**	**0.0039**	**0.0130**	x

P-values of the pairwise comparison of the ROC curves calculated with roccomp of STATA.

Abbreviations: BISEP, Burden of illness Score for Elderly Persons; ROC, receiver operating characteristic

© 2022 Schneider C et al. *JAMA Network Open.*

eTable 4. Calibration of the Six Scores Analyzed With the Brier Score and the Hosmer-Lemeshow Chi Square Test With the Hosmer-Lemeshow Chi Square Test Run on a Subsample

Score	Predicted mortality		Observed mortality		Brier	Hosmer-Lemeshow		HL subsample*	
	%	95% CI	%	95% CI		Chi^2	p-value	Chi^2	p-value
BISEP	8<br>24<br>51<br>74		10<br>14<br>23<br>30	(8-13)<br>(11-18)<br>(19-27)<br>(26-34)	0.24	767.5	< 0.01	39.3	< 0.001
CARING Criteria	<18<br>18-48<br>≥49		14<br>21<br>30	(12-16)<br>(17-25)<br>(26-34)	0.17	104.7	< 0.01	7.5	0.06
Charlson Comorbidity Index	12<br>26<br>52<br>85		10<br>15<br>24<br>31	(7-15)<br>(13-18)<br>(20-27)<br>(26-37)	0.23	938.4	< 0.01	37.4	< 0.01
Gagné Index	2.4<br>3.6<br>5.1<br>7.8<br>11.3<br>14.6<br>20.1<br>24.9<br>29.5<br>36.5<br>46.8	(2.2-2.6)<br>(3.4-3.8)<br>(4.9-5.4)<br>(7.4-8.3)<br>(10.7-12.0)<br>(13.8-15.5)<br>(18.9-21.4)<br>(23.3-26.5)<br>(24.4-31.6)<br>(34.4-38.7)<br>(43.4-50.1)	7.7<br>11.2<br>15.4<br>20.1<br>18.1<br>21.8<br>31.4<br>37.0<br>32.1<br>44.9<br>54.2	(3.9-13.4)<br>(7.9-15.3)<br>(11.7-19.7)<br>(15.5-25.3)<br>(13.5-23.7<br>(16.0-27.9)<br>(24.3-39.3)<br>(26.6-48.5)<br>(20.3-46.0)<br>(30.7-59.8)<br>(32.8-74.4)	0.16	89.8	< 0.01	1.2	1.00
Levine Index	14<br>18<br>32<br>46	(11-16)<br>(15-21)<br>(28-36)<br>(42-50)	3<br>6<br>14<br>27	(0-15)<br>(3-10)<br>(11-17)<br>(24-30)	0.19	271.7	< 0.01	21.6	< 0.01
Walter Index	13<br>20<br>37<br>68	(10-16)<br>(16-24)<br>(33-41)<br>(63-73)	6<br>15<br>22<br>39	(4-9)<br>(12-18)<br>(19-25)<br>(34-45)	0.17	206.8	< 0.01	12.2	0.02

Abbreviations: BISEP, Burden of illness Score for Elderly Persons; CI, confidence interval; HL Hosmer-Lemeshow.
* Calibration assessed in a random selected subsample of 100 participants.

© 2022 Schneider C et al. *JAMA Network Open.*

eTable 5. Sensitivity Analysis. Overall performance, discriminatory ability and calibration of the six scores analyzed with the Brier score, C-statistics and Hosmer-Lemeshow goodness of fit test using multiple imputation for missing data in variables for albumin values and nursing home residence.

**Score**	**Overall performance[1]** Brier score	**Discriminatory ability**		**Calibration** Hosmer-Lemeshow[2]	
		C-Statistic	95% CI	Chi^2	p-value
**BISEP**	0.25	0.65	(0.62-0.68)	927.6	< 0.01
**CARING Criteria**	0.18	0.64	(0.61-0.67)	201.3	< 0.01
**Charlson Index**	0.23	0.62	(0.59-0.65)	938.4	< 0.01
**Gagné Index**	0.16	0.65	(0.62-0.68)	89.8	< 0.01
**Levine Index**	0.19	0.66	(0.63-0.69)	271.7	< 0.01
**Walter Index**	0.17	0.69	(0.66-0.72)	209.9	< 0.01

Abbreviations: AUC, area under the receiver operating characteristic curve; BISEP, Burden of illness Score for Elderly Persons; CI, confidence interval.

[1] The Brier score ranges from 0 (perfect overall performance) to 0.25 (non-informative model).

[2] The null hypothesis of the HL goodness of fit test is that the assessed score predicts death correctly. Thus a significant p-value indicates a poor calibration.

© 2022 Schneider C et al. *JAMA Network Open.*

eTable 6: Sensitivity Analysis. Overall Performance, Discriminatory Ability and Calibration of the Six Scores Analyzed With the Brier Score, C-statistics and Hosmer-Lemeshow Goodness of Fit Test After Excluding All 71 Death During Hospitalization

**Score**	**Overall performance**[1] Brier score	**Discriminatory ability**		**Calibration** Hosmer-Lemeshow[2]	
		C-Statistic	95% CI	Chi^2	p-value
**BISEP**	0.24	0.64	(0.61-0.67)	894.3	< 0.01
**CARING Criteria**	0.16	0.65	(0.62-0.69)	161.9.7	< 0.01
**Charlson Index**	0.23	0.62	(0.59-0.66)	1066.2	< 0.01
**Gagné Index**	0.14	0.65	(0.62-0.69)	43.0	< 0.01
**Levine Index**	0.18	0.66	(0.63-0.69)	355.4	< 0.01
**Walter Index**	0.17	0.69	(0.66-0.72)	280.7	< 0.01

Abbreviations: AUC, area under the receiver operating characteristic curve; BISEP, Burden of illness Score for Elderly Persons; CI, confidence interval.

[1] The Brier score ranges from 0 (perfect overall performance) to 0.25 (non-informative model).

[2] The null hypothesis of the HL goodness of fit test is that the assessed score predicts death correctly. Thus a significant p-value indicates a poor calibration.

© 2022 Schneider C et al. *JAMA Network Open.*

eTable 7. OPERAM Patient Characteristics at Baseline With Information About Missing Data

	Study population (N = 1879)	
		Number of participants with missing data, n (%)
**Female, n (%)**	835 (44.4)	0
**Male, n (%)**	1044 (55.6)	0
**Age (years), median (IQR)**	79 (74-84)	0
**Length of hospital stay (days), median (IQR)**	8.5 (6-14)	45 (2.4)
**Number of diagnoses, median (IQR)**	11 (8-16)	0
**Number of drugs, median (IQR)**	11 (8-14)	0
**Current or experienced Cancer, n (%)**	520 (27.7)	0
**Living in nursing home, n (%)**	96 (5.9)	226 (12.0)
**Discharge to nursing home, n (%)**	155 (8.4)	30 (1.6)
**≥2 admissions in last year, n (%)**	445 (23.8)	6 (0.3)
**Laboratory, median (IQR)**		
**Albumin (g/l)**	33 (28-37)	717 (38.2)
**Creatinine (µmol/l)**	95 (74-128)	22 (1.2)
**Activity of daily living, n (%)**		
**Dependent for bathing**	596 (32.0)	18 (1.0)
**Dependent for feeding**	150 (8.0)	9 (0.5)
**Dependent for dressing**	524 (28.0)	8 (0.4)
**Dependent for toileting and hygiene**	338 (18.1)	9 (0.5)
**Dependent for transferring**	329 (17.6)	8 (0.4)
**Dependent for mobility**	405 (21.7)	10 (0.5)
**Study site, n (%)**		
**Switzerland**	805 (42.8)	
**Belgium**	338 (18.0)	
**The Netherlands**	406 (21.6)	
**Republic of Ireland**	330 (17.6)	

Abbreviations: IQR, interquartile range; n, number.
Conversion factor to convert from International System of Units (SI) into conventional units: albumin 0.1, creatinine 0.0113.

© 2022 Schneider C et al. *JAMA Network Open.*

References

1. Barnett K, Mercer SW, Norbury M, Watt G, Wyke S, Guthrie B. Epidemiology of multimorbidity and implications for health care, research, and medical education: a cross-sectional study. *Lancet*. Jul 7 2012;380(9836):37-43. doi:10.1016/S0140-6736(12)60240-2
2. Benetos A, Rossignol P, Cherubini A, et al. Polypharmacy in the Aging Patient: Management of Hypertension in Octogenarians. *Jama*. Jul 14 2015;314(2):170-80. doi:10.1001/jama.2015.7517
3. Gueyffier F, Bulpitt C, Boissel JP, et al. Antihypertensive drugs in very old people: a subgroup meta-analysis of randomised controlled trials. INDANA Group. *Lancet*. Mar 6 1999;353(9155):793-6.
4. Boyd CM, Darer J, Boult C, Fried LP, Boult L, Wu AW. Clinical practice guidelines and quality of care for older patients with multiple comorbid diseases: implications for pay for performance. *Jama*. Aug 10 2005;294(6):716-24. doi:10.1001/jama.294.6.716
5. Payne RA, Abel GA, Avery AJ, Mercer SW, Roland MO. Is polypharmacy always hazardous? A retrospective cohort analysis using linked electronic health records from primary and secondary care. *British journal of clinical pharmacology*. Jun 2014;77(6):1073-82. doi:10.1111/bcp.12292
6. Lee SJ, Leipzig RM, Walter LC. Lag time to benefit for preventive therapies--reply. *Jama*. Apr 16 2014;311(15):1567-8. doi:10.1001/jama.2014.2325
7. Iyer S, Naganathan V, McLachlan AJ, Le Couteur DG. Medication withdrawal trials in people aged 65 years and older: a systematic review. *Drugs & aging*. 2008;25(12):1021-31. doi:10.2165/0002512-200825120-00004
8. Yourman LC, Lee SJ, Schonberg MA, Widera EW, Smith AK. Prognostic indices for older adults: a systematic review. *Jama*. Jan 11 2012;307(2):182-92. doi:10.1001/jama.2011.1966
9. Moons KG, Altman DG, Reitsma JB, et al. Transparent Reporting of a multivariable prediction model for Individual Prognosis or Diagnosis (TRIPOD): explanation and elaboration. *Annals of internal medicine*. Jan 6 2015;162(1):W1-73. doi:10.7326/M14-0698
10. Adam L, Moutzouri E, Baumgartner C, et al. Rationale and design of OPtimising thERapy to prevent Avoidable hospital admissions in Multimorbid older people (OPERAM): a cluster randomised controlled trial. *BMJ open*. Jun 3 2019;9(6):e026769. doi:10.1136/bmjopen-2018-026769
11. Blum MR, Sallevelt B, Spinewine A, et al. Optimizing Therapy to Prevent Avoidable Hospital Admissions in Multimorbid Older Adults (OPERAM): cluster randomised controlled trial. *Bmj*. Jul 13 2021;374:n1585. doi:10.1136/bmj.n1585
12. Inouye SK, Bogardus ST, Jr., Vitagliano G, et al. Burden of illness score for elderly persons: risk adjustment incorporating the cumulative impact of diseases, physiologic abnormalities, and functional impairments. *Medical care*. Jan 2003;41(1):70-83. doi:10.1097/01.MLR.0000039829.60382.12
13. Fischer SM, Gozansky WS, Sauaia A, Min SJ, Kutner JS, Kramer A. A practical tool to identify patients who may benefit from a palliative approach: the CARING criteria. *Journal of pain and symptom management*. Apr 2006;31(4):285-92. doi:10.1016/j.jpainsymman.2005.08.012
14. Charlson ME, Pompei P, Ales KL, MacKenzie CR. A new method of classifying prognostic comorbidity in longitudinal studies: development and validation. *Journal of chronic diseases*. 1987;40(5):373-83.
15. Gagne JJ, Glynn RJ, Avorn J, Levin R, Schneeweiss S. A combined comorbidity score predicted mortality in elderly patients better than existing scores. *Journal of clinical epidemiology*. Jul 2011;64(7):749-59. doi:10.1016/j.jclinepi.2010.10.004
16. Levine SK, Sachs GA, Jin L, Meltzer D. A prognostic model for 1-year mortality in older adults after hospital discharge. *The American journal of medicine*. May 2007;120(5):455-60. doi:10.1016/j.amjmed.2006.09.021
17. Walter LC, Brand RJ, Counsell SR, et al. Development and validation of a prognostic index for 1-year mortality in older adults after hospitalization. *Jama*. Jun 20 2001;285(23):2987-94.
18. Quan H, Sundararajan V, Halfon P, et al. Coding algorithms for defining comorbidities in ICD-9-CM and ICD-10 administrative data. *Medical care*. Nov 2005;43(11):1130-9.
19. Fine MJ, Auble TE, Yealy DM, et al. A prediction rule to identify low-risk patients with community-acquired pneumonia. *The New England journal of medicine*. Jan 23 1997;336(4):243-50. doi:10.1056/nejm199701233360402
20. Rufibach K. Use of Brier score to assess binary predictions. *Journal of clinical epidemiology*. Aug 2010;63(8):938-9; author reply 939. doi:10.1016/j.jclinepi.2009.11.009
21. Hanley JA, McNeil BJ. The meaning and use of the area under a receiver operating characteristic (ROC) curve. *Radiology*. Apr 1982;143(1):29-36. doi:10.1148/radiology.143.1.7063747
22. DeLong ER, DeLong DM, Clarke-Pearson DL. Comparing the areas under two or more correlated receiver operating characteristic curves: a nonparametric approach. *Biometrics*. Sep 1988;44(3):837-45.
23. Nattino G, Pennell ML, Lemeshow S. Assessing the goodness of fit of logistic regression models in large samples: A modification of the Hosmer-Lemeshow test. *Biometrics*. Jun 2020;76(2):549-560. doi:10.1111/biom.13249
24. D'Agostino RB, Sr., Vasan RS, Pencina MJ, et al. General cardiovascular risk profile for use in primary care: the Framingham Heart Study. *Circulation*. Feb 12 2008;117(6):743-53. doi:10.1161/CIRCULATIONAHA.107.699579

© 2022 Schneider C et al. *JAMA Network Open.*

25. Bannay A, Chaignot C, Blotiere PO, et al. The Best Use of the Charlson Comorbidity Index With Electronic Health Care Database to Predict Mortality. *Medical care*. Feb 2016;54(2):188-94. doi:10.1097/MLR.0000000000000471
26. Monacelli F, Tafuro M, Molfetta L, et al. Evaluation of prognostic indices in elderly hospitalized patients. *Geriatrics & gerontology international*. Jun 2017;17(6):1015-1021. doi:10.1111/ggi.12801
27. Sommers BD, Gawande AA, Baicker K. Health Insurance Coverage and Health - What the Recent Evidence Tells Us. *The New England journal of medicine*. Aug 10 2017;377(6):586-593. doi:10.1056/NEJMsb1706645
28. Wilper AP, Woolhandler S, Lasser KE, McCormick D, Bor DH, Himmelstein DU. Health insurance and mortality in US adults. *American journal of public health*. Dec 2009;99(12):2289-95. doi:10.2105/AJPH.2008.157685
29. Woolhandler S, Himmelstein DU. The Relationship of Health Insurance and Mortality: Is Lack of Insurance Deadly? *Annals of internal medicine*. Sep 19 2017;167(6):424-431. doi:10.7326/M17-1403
30. Bertolini G, D'Amico R, Nardi D, Tinazzi A, Apolone G. One model, several results: the paradox of the Hosmer-Lemeshow goodness-of-fit test for the logistic regression model. *Journal of epidemiology and biostatistics*. 2000;5(4):251-3.
31. Kramer AA, Zimmerman JE. Assessing the calibration of mortality benchmarks in critical care: The Hosmer-Lemeshow test revisited. *Critical care medicine*. Sep 2007;35(9):2052-6. doi:10.1097/01.ccm.0000275267.64078.b0
32. Steyerberg EW. *Clinical Prediction Models*. Springer 2010.
33. Paul P, Pennell ML, Lemeshow S. Standardizing the power of the Hosmer-Lemeshow goodness of fit test in large data sets. *Stat Med*. Jan 15 2013;32(1):67-80. doi:10.1002/sim.5525

© 2022 Schneider C et al. *JAMA Network Open.*
